# Comparisons of protein profiles of beech bark disease resistant and susceptible American beech (*Fagus grandifolia*)

**DOI:** 10.1186/1477-5956-11-2

**Published:** 2013-01-14

**Authors:** Mary E Mason, Jennifer L Koch, Marek Krasowski, Judy Loo

**Affiliations:** 1 Department of Entomology, Ohio Agricultural Research and Development Center, The Ohio State University, 1680 Madison Ave, Wooster, OH, 44691, USA; 2 US Forest Service, Northern Research Station, 359 Main Rd, Delaware, OH, 43015, USA; 3 Faculty of Forestry and Environmental Management, University of New Brunswick, P.O. Box 4400, 28 Dineen Drive, Fredericton, NB, E3B 5A3, Canada; 4 Formerly: Canadian Forest Service, Natural Resources Canada, P.O. Box 4000, Fredericton, NB, E3B 6J6, Canada; 5 Present address: Bioversity International, Via dei Tre Denari, 472/a, 00057 Maccarese, Rome, Italy

**Keywords:** Beech bark disease, Beech scale, Disease resistance, Insect resistance, Fagus, Cryptococcus, Neonectria

## Abstract

**Background:**

Beech bark disease is an insect-fungus complex that damages and often kills American beech trees and has major ecological and economic impacts on forests of the northeastern United States and southeastern Canadian forests. The disease begins when exotic beech scale insects feed on the bark of trees, and is followed by infection of damaged bark tissues by one of the *Neonectria* species of fungi. Proteomic analysis was conducted of beech bark proteins from diseased trees and healthy trees in areas heavily infested with beech bark disease. All of the diseased trees had signs of *Neonectria* infection such as cankers or fruiting bodies. In previous tests reported elsewhere, all of the diseased trees were demonstrated to be susceptible to the scale insect and all of the healthy trees were demonstrated to be resistant to the scale insect. Sixteen trees were sampled from eight geographically isolated stands, the sample consisting of 10 healthy (scale-resistant) and 6 diseased/infested (scale-susceptible) trees.

**Results:**

Proteins were extracted from each tree and analysed in triplicate by isoelectric focusing followed by denaturing gel electrophoresis. Gels were stained and protein spots identified and intensity quantified, then a statistical model was fit to identify significant differences between trees. A subset of BBD differential proteins were analysed by mass spectrometry and matched to known protein sequences for identification. Identified proteins had homology to stress, insect, and pathogen related proteins in other plant systems. Protein spots significantly different in diseased and healthy trees having no stand or disease-by-stand interaction effects were identified.

**Conclusions:**

Further study of these proteins should help to understand processes critical to resistance to beech bark disease and to develop biomarkers for use in tree breeding programs and for the selection of resistant trees prior to or in early stages of BBD development in stands. Early identification of resistant trees (prior to the full disease development in an area) will allow forest management through the removal of susceptible trees and their root-sprouts prior to the onset of disease, allowing management and mitigation of costs, economic impact, and impacts on ecological systems and services.

## Background

Beech bark disease (BBD) is an insect-fungus complex that has been killing American beech (*Fagus grandifolia* Ehrh.*)* trees since the accidental introduction of the beech scale insect (*Cryptococcus fagisuga* Lind.) to Canada around 1890 [1,2]. The first phase of BBD is beech scale insect infestation resulting in the production of small fissures in the bark [1]. The fungal component, either *Neonectria ditissima* Samuels & Rossman or *Neonectria faginata* Castlebury then infects these fissures causing extensive tissue damage. Mortality in the first wave of the disease can be as high as 50 % [3], with consequent loss to stand health, merchantable timber, and many wildlife and ecosystem services.

An estimated 1% of American beech trees remain disease free in forests long-affected by beech bark disease [4]. Insect challenge experiments have demonstrated that resistance is to the beech scale portion of the disease complex [4,5]. Although it has been reported that scale infestation without *Neonectria* infection may play a role in mortality events [6], there has been no documentation of *Neonectria* infection leading to widespread stand or landscape level mortality in the absence of prior scale infestation. In aftermath forests, where scale populations have declined, presumably due to loss or reduced quality of habitat [7] and environmental factors [8], the population dynamics of scale and *Neonectria* are no longer directly correlated [7,9]. However, in these cases it is suggested that the lower density of scale is still sufficiently high that infection sites (scale-feeding wounds) are not limiting and once the fungus has established in the tree it is no longer influenced by fluctuations in scale density [7]. Therefore, the focus of recent breeding and tree improvement efforts has been on resistance to the scale insect [5,10]. Current management approaches are based on the objective of increasing the proportion of disease resistant beech by removing susceptible trees along with any resulting root and stump sprouts, retaining the disease-free trees prior to the height of BBD development [11,12] and supplementing with genetically scale-resistant seedling plantings once such materials are available (management plans of US Forest Service Allegheny National Forest [13] and Michigan Dept. of Natural Resources [14]). However, it is impossible to identify the most resistant beech trees until the scale infestation is heavy, at which point economic losses have already occurred and management operations are more complicated and expensive. Identification of a biomarker for *Cryptococcus* resistance would provide land managers the opportunity to begin management operations before the economic and ecological losses have occurred, and to spread BBD management activities over several budget cycles. A biomarker for resistance may also be utilized to expedite the breeding and selection process.

Bark protein differences are likely to be a good source for biomarker candidates. The scale insect feeds in the tissue layers vernacularly known as bark (cork, cork cambium, phloem, and cambium) [1]. Wargo et al. [15] found significant differences in bark amino acid concentrations and total amino nitrogen in different seasons and scale infestation levels. These results suggest bark protein content may be important in the insect-tree interaction, and that bark protein profiles may differ between scale-resistant and susceptible beech trees. Therefore differentially expressed bark proteins may be reliable biomarkers of resistance to beech scale in American beech. One method to identify differentially expressed bark proteins is to examine the proteome of a number of trees using two dimensional electrophoresis (2-DE) gels. A proteomics approach allows the examination and quantification of large numbers of proteins anonymously and simultaneously. Typically, 2-DE analysis is limited to two-sample comparison with simple experimental structure (e.g. one treatment). Utilization of analysis of variance (ANOVA) for statistical analysis allows the testing of three or more treatment levels for several technical and biological factors in one model, and supports unbalanced experimental and sampling designs. This more sophisticated analysis allows the identification of more complex protein quantity patterns and the interactions of factors in protein quantity. In this study we employ the use of 2-DE gel based proteomics and ANOVA to identify proteins in the bark of American beech that are different between healthy and BBD diseased trees, while also considering if the BBD effect is present alone or with a stand effect or interaction between stand and BBD effects. Although the healthy trees in this study are known to be resistant to the scale insect, the diseased trees are susceptible to both scale infestation (Table 1), and had symptoms of an active fungal infection at the time of tissue collection. Proteins that are expressed in response to the scale insect cannot be distinguished from proteins expressed in response to the fungal pathogen in diseased trees so in our analysis we refer to the more general BBD response which includes responses to both. This approach allows selection of proteins for further study that are most likely to be broadly linked to BBD response rather than different in protein quantity due to the relatedness of trees within stands.

**Table 1:** Tree information including stand, field disease score, controlled test insect challenge result, and spot quantities

**Tree name**	**Stand code**^**a**^	**Disease Condition**^**b**^	**Scale Interaction**^**c**^	**Total protein spots**	**Number of matched spots**	**Number of unique spots**
1504	LM	H	R (8)	382	295	87
1506	LM	D	S (3)	197	184	13
CL01^d^	CL	H	R (10)	258	241	17
CM01	CM	H	R (1)	280	270	10
CM02D^d^	CM	D	S(11)	329	329	0
DT02^d^	DT	H	R (2)	228	215	13
DT02D	DT	D	not tested	286	266	20
DTOA^d^	DT	H	R (2)	522	421	101
JC01^d^	JC	H	R (14)	367	338	29
JC02D	JC	D	S (4)	487	408	79
OD02^d^	OD	H	R (10)	284	272	12
OD08D^d^	OD	D	S (12)	239	225	14
RS01^d^	RS	H	R (9)	288	265	23
RS02D	RS	D	S (7)	229	223	6
SL01^d^	SL	H	R (4)	249	240	15
SL03^d^	SL	H	R (8)	260	243	17

^a^LM=Ludington, MI, USA; CL= Charlie Lake, NB, Can; CM= Curry Mountain, NB, Can; DT= Roachville, NB, Can; JC=Highfield, NB, Can; OD= Odell Park, Fredericton, NB, Can; RS= Regent St., Fredericton, NB, Can; SL=Spednick Lake, NB, Can.

^b^H=Healthy (lacking scale infestation and signs of fungal infection), D= Diseased (beech bark disease present both scale infestation and fungal infection observed (on ortet) in the field).

^c^Test result of artificial inoculation of grafted ramets with scale insect eggs. Result (number of ramets tested, i.e. replicates). R= Resistant, 0–5 avg. live scale, S= Susceptible, 30–100 avg. live scale and expected to sustain scale population and subsequent fungal infection.

^d^Correspondence to Ramirez et al. [16] CL01=E1, CM02d=H1, DT02=B3, DT0A=B1, JC01=C1, OD02=A2, OD08d=A3, RS01=I1, SL01=D1, and SL03=D3.

## Results

### Individual tree analysis and spot matching

**Figure 1:**
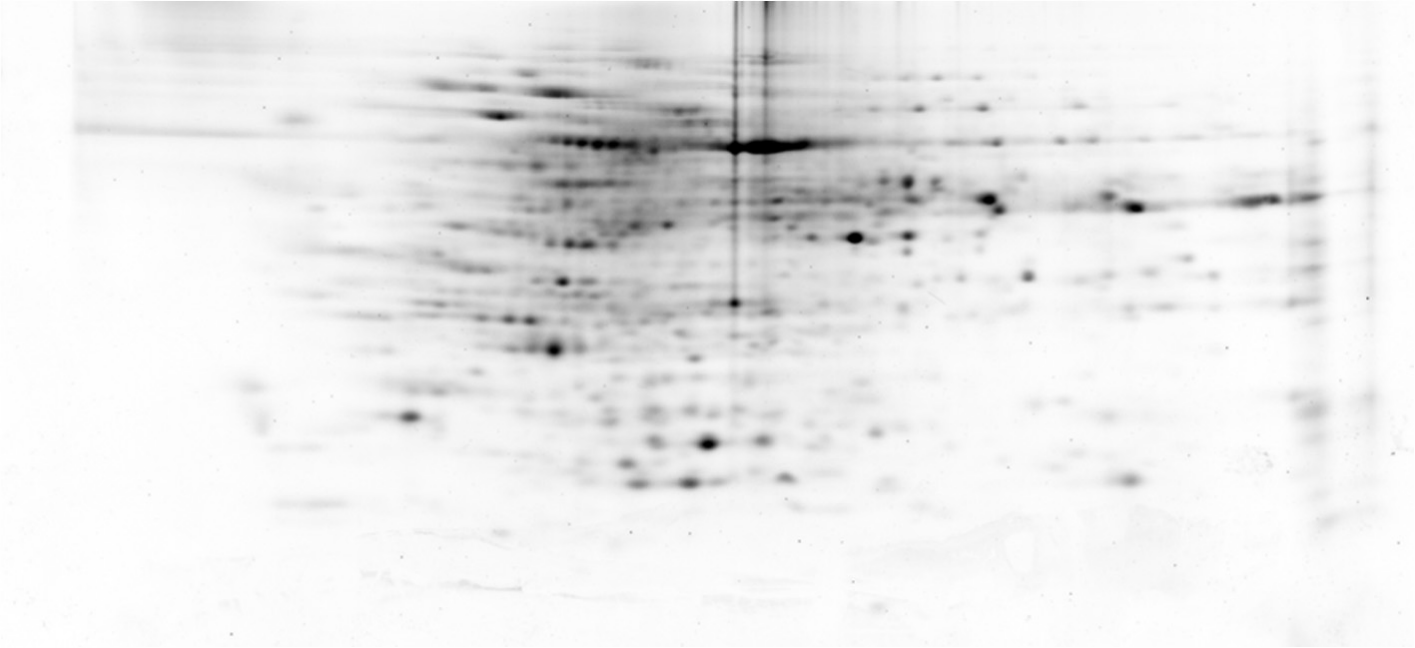
**Raw gel image of one randomly selected gel.** Gel image from tree DT02, shown to illustrate the number of spots and quality of separation typical of the experimental gels. The gel has been cropped to remove gel edge and markers, but include both pI and electrophoresis fronts.

The location, field disease score, and the artificial infestation (scale resistant/scale susceptible) results for the ten healthy trees and six diseased trees studied are summarized in Table 1. Artificial infestation of grafted ramets of the healthy trees demonstrated that all of these genotypes are resistant to the scale insect, the details of these experiments are reported elsewhere [16]. Protein was extracted and 2-DE was conducted for three technical replicates per tree. Figure 1 shows a randomly selected experimental gel to illustrate the typical resolution and spot density we achieved in the experiment. PDQuest was used to create a master gel for each tree and the number of protein spots per tree ranged from 197 to 522 with an average of 305.3 and standard error of 23.

**Figure 2:**
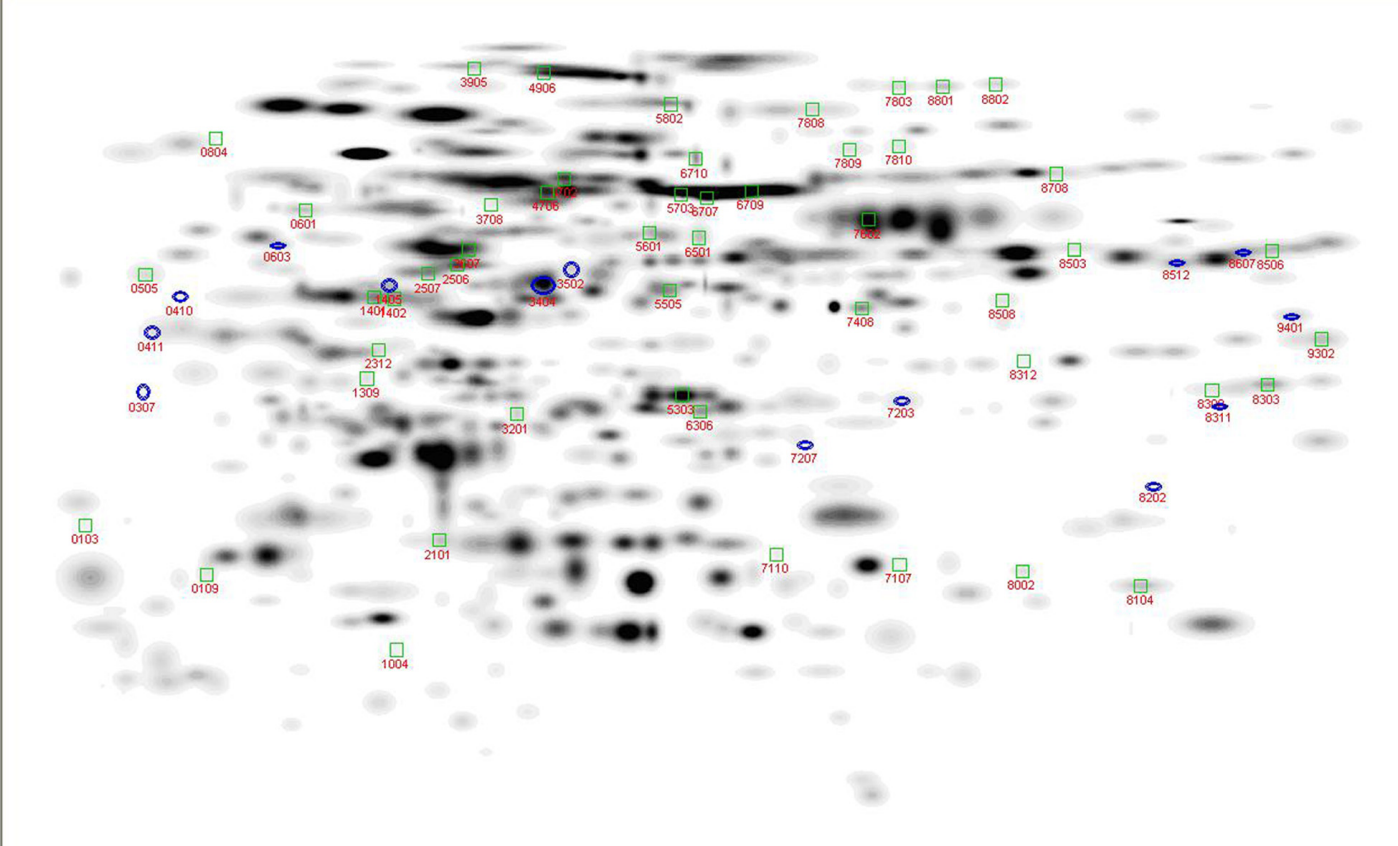
**Global master gel showing the Gaussian spots from all experimental gels.** Spots identified as having a significant BBD effect are marked and numbered, with a blue circle for spots that are higher in healthy trees (compared to BBD diseased trees), and a green box for spots that are lower in healthy trees . The image is a composite of the full 48 gels so weaker spots may not be visible in the image (but are present and can be seen at higher magnification and contrast).

An experiment-wide master gel was constructed using the 'compare experiments' function of PDQuest where each individual tree master was considered an 'experiment'. The experiment wide master gel (Figure 2) included all spots on individual tree masters that were added to the experiment wide master because they were present in two or more trees. Most, but not all, of these spots were present in more than two trees, and some spots were present in all trees. The number of matched and unique spots for each tree is listed in Table 1. The total number of spots added to the experiment wide master (matched in two or more trees) was 531. Matched spot per tree ranged from 184 to 421 and average 277.2 +/− 16.7. Unique spots per tree (spots in the tree master not matched and added to the experiment wide master) range from 0 (base tree CM02d has all spots on the master gel by definition) to 101 with an average of 28.5 +/− 7.75. The grand total of spots identified is 987 which is the sum of the 531 matched spots plus 456 spots unique to only one tree. Spots unique to a single tree were excluded from further analysis. While unique spots in aggregate are 46% of total spots, a maximum of 22.7% of the spots in any one tree are unique indicating that the spot matching of trees to the experiment wide master was efficient.

To consider whether these unique spots could be artifacts due to low spot intensity, four trees were selected at random and the spot intensity distribution examined. Graphing showed that the distribution of unique spots was slightly biased toward lighter spots. However, comparison of summary statistics (mean, min, Q1, mean, Q3, max) illustrates that spots of similar intensity are both matched and unmatched, and the faintest spot is matched in three of the four trees (data not shown). This indicates that the spots unique to each tree are not artifacts of poor matching related to the intensity of the spots or variances in protein quantification. Spot quantities for all matched spots in all trees were exported for analysis (missing spots were estimated) in order to use more robust statistical procedures than PDQuest allows.

### Constitutive proteins and technical effects

General linear models were fitted to assess whether technical effects (gel and batch) were significant or could be ignored, and to identify constitutive proteins (spot quantity equal in all individual trees, tested by fitting a tree effect). Technical effects were significant for only six spots and these spots were dropped from further analysis, allowing technical effects to be dropped from later models. The biological effects of interest are stand and disease state, and constitutive spots will obviously not vary for these effects. Therefore the 103 constitutive spots (tree effect not significant) were removed from the dataset to reduce its size for more efficient analysis (leaving 422 spots that had a significantly different mean spot quantity in at least one tree and no significant technical effects).

### Stand and disease condition effects

General linear models were fitted to test for disease condition effect (BBD), stand (STAND) and the disease condition by stand interaction (INT) for the retained spots. The count of spots significant for different combinations of effects is shown in Table 2. The largest class of proteins contains those that are quantitatively different in several trees (demonstrated in the first model, but not significant for any tested effects (209 spots)). Protein spots with a significant STAND effect but no significance for BBD (61 spots of which 28 have a significant interaction and 33 do not) are interesting as potential markers for geographic variation in beech. These spots and an additional 32 spots with only the interaction effect found significant were not studied further in this experiment.

**Table 2:** Significant protein counts

	**Stand x BBD Interaction**			
	**Significant**		**Not Significant**	
**STAND effect**	**Significant**	**Not Sig.**	**Significant**	**Not Sig.**
BBD Effect Significant	47	9	14	50
BBD Effect Not Significant	28	32	33	209

**Figure 3:**
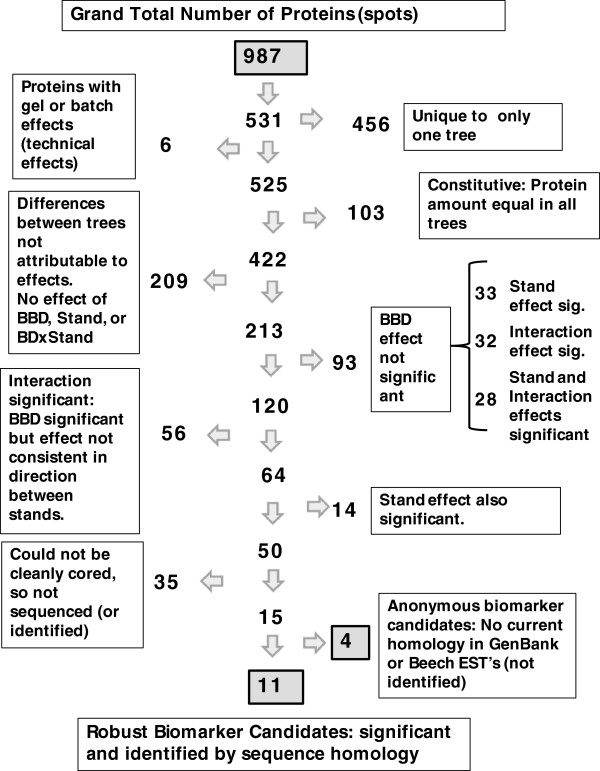
**Flow chart illustrating the reduction in the number of protein spots of interest using our experimental and statistical approach.** The flow chart shows the starting number of protein spots and how many of the spots were eliminated from further consideration at each step or decision point. The dramatic reduction in the number of spots of interest to only 11 spots demonstrates the discriminatory power of the experimental and statistical approach.

Count of proteins with significant effects for stand, beech bark disease state, and stand x disease state interaction. The most likely biomarker candidates are the protein spots with a significant BBD effect in the absence of stand effects and interactions. To see the correspondence to Figure 3 may require summing across cells, for example the 56 spots where the interaction and BBD effect are significant is the sum of the two stand levels, 47 plus nine.

Table 3 shows the protein spots with significant BBD effect, including the p-value and q-value for the BBD effect, the mean spot quantity and standard error for the diseased and healthy trees, and the ratio and direction of differences of spot quantity of healthy to diseased to trees (for all p-values underlying Table 3, see Additional file 1). One-hundred and twenty spots have a significant BBD effect, and of these 50 have no other significant effects and are the most logical candidates for biomarker development (marked with an asterisk in Table 3).

**Table 3:** Significant effects, means, and expression ratio for protein spots with significant beech bark disease (BBD) effect

**Spot**		**BBD q-value**^**a**^	**STAND**^**b**^	**INT**^**c**^	**mean_H**^**d**^	**sd_H**^**d**^	**mean_D**^**d**^	**sd_D**^**d**^	**Ratio of means**^**e**^	**Direction in healthy**
11		0.0470	NS	SIG	26,174	65,030	72,775	87,910	2.7804	down
13		0.0328	SIG	SIG	94,894	219,401	59,736	143,489	1.5886	up
19		0.0000^f^	SIG	SIG	8,004	5,915	62,232	97,523	7.7749	down
103	*	0.0199	NS	NS	55,653	177,669	296,935	581,023	5.3354	down
108		0.0004	SIG	SIG	258,261	376,114	111,723	168,407	2.3116	up
109	*	0.0023	NS	NS	617,595	986,852	153,958	248,739	4.0114	up
110		0.0001	SIG	SIG	39,356	97,166	220,462	387,308	5.6017	down
210		0.0004	NS	SIG	757,282	605,616	285,096	446,196	2.6562	up
303		0.0002	SIG	SIG	173,237	616,608	633,878	976,099	3.6590	down
306		0.0022	NS	SIG	8,004	5,915	149,834	274,377	18.7195	down
307		0.0275	SIG	NS	759,264	688,274	302,438	516,028	2.5105	up
408		0.0000^f^	SIG	SIG	258,525	539,218	856,239	1,833,144	3.3120	down
410		0.0041	SIG	NS	26,580	63,389	118,326	141,769	4.4517	down
411		0.0295	SIG	NS	165,226	431,575	708,030	1,274,849	4.2852	down
412		0.0054	SIG	SIG	447,495	1,004,238	310,856	722,114	1.4396	up
505	*	0.0470	NS	NS	925,741	886,816	477,691	544,289	1.9379	up
601	*	0.0318	NS	NS	333,862	424,610	725,308	843,336	2.1725	down
602		0.0000^f^	SIG	SIG	365,242	873,890	1,394,639	1,721,661	3.8184	down
603		0.0076	SIG	NS	1,533,077	1,657,207	1,053,773	1,360,574	1.4548	up
606		0.0216	SIG	SIG	43,523	126,690	8,417	8,283	5.1708	up
610		0.0001	SIG	SIG	146,957	447,440	8,417	8,283	17.4593	up
704		0.0000^f^	SIG	SIG	68,064	212,603	899,841	2,069,461	13.2205	down
804	*	0.0013	NS	NS	8,004	5,915	148,985	250,618	18.6134	down
1004	*	0.0023	NS	NS	8,004	5,915	175,554	294,631	21.9328	down
1005		0.0012	SIG	SIG	83,946	197,401	354,800	440,759	4.2265	down
1109		0.0000^f^	SIG	SIG	83,319	265,430	503,759	1,143,792	6.0462	up
1303		0.0012	SIG	SIG	364,487	1,135,686	155,251	282,766	2.3477	up
1309	*	0.0043	NS	NS	297,936	428,232	70,605	95,878	4.2197	up
1401	*	0.0075	NS	NS	1,381,518	1,428,751	573,957	882,187	2.4070	up
1402	*	0.0168	NS	NS	1,221,781	1,194,937	2,454,219	2,386,797	2.0087	down
1405		0.0219	SIG	NS	536,821	589,544	164,445	364,867	3.2644	up
1704		0.0076	NS	SIG	107,476	344,505	651,980	1,109,723	6.0663	down
2101	*	0.0238	NS	NS	1,068,400	1,220,098	824,152	883,156	1.2964	up
2107		0.0158	SIG	SIG	65,274	230,953	293,355	519,761	4.4942	down
2312	*	0.0284	NS	NS	350,710	704,649	8,417	8,283	41.6664	up
2506	*	0.0469	NS	NS	1,897,806	1,096,881	2,497,681	1,221,943	1.3161	down
2507	*	0.0012	NS	NS	417,660	599,615	1,048,242	1,050,906	2.5098	down
2611		0.0498	SIG	SIG	1,535,001	1,783,491	2,205,570	2,796,718	1.4369	down
2614		0.0000^f^	SIG	SIG	8,004	5,915	285,838	432,421	35.7112	down
2615		0.0008	SIG	SIG	62,288	208,601	431,929	1,018,078	6.9344	down
2801		0.0000^f^	SIG	SIG	8,004	5,915	869,550	1,285,546	108.6371	down
2803		0.0010	SIG	SIG	712,668	1,115,376	580,029	825,345	1.2287	up
2806		0.0001	SIG	SIG	443,822	1,084,784	129,571	442,770	3.4253	up
3201	*	0.0016	NS	NS	1,336,983	1,205,431	794,583	908,778	1.6826	up
3305		0.0000^f^	SIG	SIG	89,683	248,936	303,148	448,565	3.3802	down
3306		0.0078	SIG	SIG	116,998	272,340	59,537	124,166	1.9651	up
3404		0.0082	SIG	NS	4,068,995	2,823,334	3,452,364	2,764,490	1.1786	up
3406		0.0000^f^	SIG	SIG	44,870	125,570	462,745	547,706	10.3130	down
3412		0.0177	NS	SIG	322,035	683,636	130,942	334,190	2.4594	up
3502		0.0188	SIG	NS	442,850	650,679	786,812	1,011,948	1.7767	down
3607	*	0.0105	NS	NS	2,247,225	2,722,559	1,430,733	2,093,163	1.5707	up
3702	*	0.0001	NS	NS	633,076	1,243,273	3,792,473	3,730,678	5.9905	down
3708	*	0.0096	NS	NS	633,632	796,621	407,271	620,398	1.5558	up
3802		0.0015	SIG	SIG	8,004	5,915	111,820	262,030	13.9703	down
3905	*	0.0039	NS	NS	8,004	5,915	137,656	242,371	17.1980	down
4106		0.0066	SIG	SIG	105,844	212,452	397,149	845,289	3.7522	down
4201		0.0010	NS	SIG	8,004	5,915	254,207	454,577	31.7593	down
4305		0.0000^f^	SIG	SIG	46,506	128,075	470,932	768,247	10.1263	down
4706	*	0.0481	NS	NS	2,739,148	3,047,552	917,692	1,062,566	2.9848	up
4802		0.0000^f^	SIG	SIG	48,748	143,131	408,963	769,834	8.3894	down
4906	*	0.0302	NS	NS	873,770	1,402,616	628,704	1,038,074	1.3898	up
5301		0.0202	SIG	SIG	450,470	363,776	399,574	418,863	1.1274	up
5303	*	0.0062	NS	NS	3,456,984	1,373,851	5,360,468	2,202,726	1.5506	down
5505	*	0.0009	NS	NS	604,226	742,627	2,522,891	2,358,561	4.1754	down
5601	*	0.0063	NS	NS	456,028	681,368	1,497,166	1,370,793	3.2831	down
5605		0.0000^f^	SIG	SIG	124,350	177,741	8,004	5,915	15.5356	up
5606		0.0000^f^	SIG	SIG	110,019	153,695	8,004	5,915	13.7452	up
5703	*	0.0297	NS	NS	3,051,574	5,434,263	478,287	1,621,642	6.3802	up
5802	*	0.0011	NS	NS	1,190,828	915,352	508,682	528,700	2.3410	up
5908		0.0010	SIG	SIG	161,910	387,868	8,004	5,915	20.2282	up
5909		0.0012	SIG	SIG	8,417	8,283	143,259	352,729	17.0200	down
6208		0.0297	SIG	SIG	186,317	276,277	80,227	222,514	2.3224	up
6303		0.0328	NS	SIG	253,951	331,973	397,930	349,663	1.5670	down
6304		0.0018	NS	SIG	2,888,828	3,401,904	820,317	1,445,597	3.5216	up
6306	*	0.0099	NS	NS	1,800,296	1,365,419	2,730,178	2,859,788	1.5165	down
6501	*	0.0318	NS	NS	1,468,936	1,183,570	604,825	1,255,327	2.4287	up
6707	*	0.0193	NS	NS	11,970,218	12,515,695	6,247,307	6,508,633	1.9161	up
6709	*	0.0238	NS	NS	8,974,280	5,564,143	5,289,975	6,857,853	1.6965	up
6710	*	0.0238	NS	NS	1,400,824	2,287,877	404,306	569,323	3.4648	up
6807		0.0070	SIG	SIG	118,758	291,241	8,004	5,915	14.8370	up
6808		0.0009	SIG	SIG	1,174,714	1,224,320	239,503	580,829	4.9048	up
6809		0.0002	SIG	SIG	376,863	587,605	95,365	213,349	3.9518	up
7009		0.0004	SIG	SIG	175,933	391,166	265,335	410,728	1.5082	down
7106		0.0000^f^	SIG	SIG	962,980	1,282,010	39,820	101,833	24.1835	up
7107	*	0.0009	NS	NS	387,387	501,608	50,505	130,300	7.6703	up
7110	*	0.0245	NS	NS	84,648	139,984	256,705	307,196	3.0326	down
7111		0.0000^f^	SIG	SIG	658,929	1,249,222	148,370	341,871	4.4411	up
7112		0.0022	NS	SIG	73,598	133,128	8,004	5,915	9.1949	up
7203		0.0261	SIG	NS	1,311,783	732,419	927,380	799,253	1.4145	up
7207		0.0186	SIG	NS	60,957	126,879	21,092	40,907	2.8901	up
7303		0.0052	SIG	SIG	246,921	602,207	615,797	1,105,070	2.4939	down
7408	*	0.0126	NS	NS	3,049,802	2,337,843	1,336,825	1,182,165	2.2814	up
7510		0.0098	SIG	SIG	305,493	558,423	397,578	1,073,627	1.3014	down
7602	*	0.0412	NS	NS	7,296,723	7,488,294	3,609,984	5,505,222	2.0213	up
7609		0.0016	SIG	SIG	381,570	946,188	8,004	5,915	47.6713	up
7803	*	0.0238	NS	NS	716,697	474,399	341,082	391,607	2.1012	up
7808	*	0.0075	NS	NS	704,023	626,350	305,591	487,526	2.3038	up
7809	*	0.0267	NS	NS	582,174	1,475,329	8,004	5,915	72.7338	up
7810	*	0.0297	NS	NS	442,246	1,119,936	8,004	5,915	55.2519	up
8002	*	0.0082	NS	NS	175,231	300,741	22,986	74,010	7.6235	up
8104	*	0.0188	NS	NS	1,096,503	874,012	394,837	541,448	2.7771	up
8202		0.0230	SIG	NS	29,407	58,674	56,069	97,123	1.9066	down
8303	*	0.0311	NS	NS	1,850,424	1,058,481	1,293,017	1,164,851	1.4311	up
8309	*	0.0008	NS	NS	340,383	449,968	902,563	716,532	2.6516	down
8311		0.0318	SIG	NS	85,387	133,171	26,149	58,170	3.2654	up
8312	*	0.0498	NS	NS	306,017	504,921	549,086	736,070	1.7943	down
8503	*	0.0193	NS	NS	587,330	907,526	123,323	558,974	4.7626	up
8506	*	0.0010	NS	NS	1,709,206	2,026,022	334,292	701,018	5.1129	up
8508	*	0.0158	NS	NS	1,395,005	1,198,636	633,833	820,628	2.2009	up
8512	*	0.0012	SIG	NS	8,417	8,283	1,173,776	1,657,190	139.4515	down
8604		0.0267	SIG	SIG	2,262,933	3,169,913	1,147,023	2,200,353	1.9729	up
8607		0.0020	SIG	NS	1,865,790	3,095,124	369,996	861,448	5.0427	up
8708	*	0.0076	NS	NS	177,743	461,377	632,706	1,403,084	3.5597	down
8801	*	0.0316	NS	NS	751,522	573,471	517,615	499,829	1.4519	up
8802	*	0.0034	NS	NS	511,552	398,150	269,962	349,834	1.8949	up
9302	*	0.0238	NS	NS	710,525	848,447	1,268,492	1,442,080	1.7853	down
9305		0.0085	SIG	SIG	301,789	462,883	368,200	614,918	1.2201	down
9401		0.0192	SIG	NS	717,956	641,915	419,853	410,107	1.7100	up
9402		0.0096	SIG	SIG	63,499	152,855	120,990	362,645	1.9054	down
9802		0.0021	SIG	SIG	112,023	263,531	8,004	5,915	13.9956	up

An asterisk to the right of the spot number indicates a spot with significant BBD effect and non-significant STAND and STANDxBBD interaction effect, and are the primary targets for biomarker development.

^a^BBD effect with q-value <0.05. q-value controls the False Discovery rate, and q<0.05 allows for no more than 26 false positives in the 425 spots tested.

^b^STAND effect with p<0.0001 (Bonferroni adjusted critical value).

^c^STANDxBBD interaction effect with p<0.0001 (Bonferroni adjusted critical value).

^d^Gaussian image spot intensity after normalization expressed in counts. Mean is the mean over the three technical replicates per tree for all trees in that disease condition. SD is standard deviation.

^e^Ratio is ratio of larger mean over smaller mean.

Mean = Gaussian image spot intensity after normalization expressed in counts. Mean is the mean over the three technical replicates for all trees in that disease condition.

SD = standard deviation.

^f^Q-values displayed as 0.0000 are positive values <0.0001. See additional table 1 for actual q-values.

### Spot selection and LC-MS/MS analysis

Spots were selected for coring and sequencing based upon the BBD effect being significant (including some with other significant effects as well) and the location of the spot in the gel being conducive to excising a clean gel core (only one protein in the core, some areas were too densely packed to allow coring). The trees 1504 and CM02d were chosen for use in preparative gels because these trees contained the most proteins of interest (33 and 26 respectively). Attempts were made to sample all BBD significant spots in these trees, and images were carefully evaluated after spot cutting to verify the intended spot was recovered for analysis. In addition to the spots of interest, several well isolated spots were cored for quality control purposes. A total of 28 gel spots (20 BBD significant, 4 control, and 4 cut from both gels) were successfully recovered and analysed by LC-MS/MS. Of the 50 highest interest spots, 15 were successfully cored and sequenced. Resulting peptide spectra were identified by matching to NCBI datasets, or in a 2-phase matching strategy matched to beech EST's that were then matched to NCBI sequences. Of the 28 spots sequenced, 20 were identified (highest scoring matches or ties only, Table 4) based upon homology to known plant sequences, or homology of the matched EST to plant sequences (including all four control spots). Of the 15 sequenced from the 50 highest interest spots, 11 were identified by sequence homology. There are a few cases where spots were matched to more than one significant identification (spots 307, 2101, and 7408), but in two of them identical peptides returned multiple database entries with different annotations (spots 307 and 2101). The use of the EST database in spot identification greatly improved the success rate at identifying proteins, as over half of the identifications were made using the EST database and would have been unidentified had only Genbank been used. The majority of the spots that were identified based on sequence homology have been shown to be stress-related in other plant systems (Table 5).

**Table 4:** Protein spots analyzed by MS/MS

**Spot** **number**	**Tree & core** **number**	**Protein ID or EST homology** **[PRIDE accession: 17706]**^**a**^	**NCBI or EST Accession** **Number**^**b**^	**Mascot** **Score**^**c**^	**Percent** **Coverage**	**Matching** **peptides**	**Peptide sequence**^**d**^	**Peptide score**^**e**^	**m/z**	**Mr(expt)**
109*^f^	1504_B6	polyphenol oxidase	ABWP2_088861_1753_2628	180	33	2	TEFAGSFVNVPSK	72.2	692.1743	1382.334
						4	GVATIGGIK	47.2	408.4638	814.913
						2	GVATIGGI**K**IQFVS	233.8	695.8282	1389.6418
						2	IQFVS	26.9	593.343	592.3357
307	1504_H5	unnamed protein product	gi|157327346	735	44	2	NILFVIS**K**PDVFK	71.3	760.6617	1519.3088
						4	SPTSDTYVIFGEAK	73.9	758.1431	1514.2716
						6	IEDLSSQLQTQAAEQFK	107.3	969.241	1936.4674
						2	DIELVMTQAGVSR	75.7	718.1302	1434.2458
						4	DIELVMTQAGVSR	98.1	709.7012	1417.3878
307	1504_H5	alpha nascent polypeptide	CCWP2_066819_3631_1428	255	52	2	NILFVIS**K**PDVFK	71.3	760.6617	1519.3088
		associated complex				4	SPTSDTYVIFGEAK	74.8	758.1431	1514.2716
						5	IEDLSSQLQTQAAEQFK	108.5	969.241	1936.4674
505	1504_A6	none	none							
1004*	CM02d_C1	thioredoxin	WOA_067025_0312_1563	120	25	2	VNTDESSSIATR	76.5	640.5275	1279.0404
						2	STLTTSIEK	46.2	490.4947	978.9748
2101*	1504_G7	ribonuclease activity regulator	CCNHS_110619_3244_2001	214	24	2	VFEDNVLVR	57.1	546.048	1090.0814
						2	VLVVDGGGSLR	67.6	537.0695	1072.1244
2101*	1504_G7	dimethylmenaquinone	gi|15232963	199	21	4	ALQPVFQIYGR	46.8	647.1753	1292.336
		methyltransferase family protein				2	VLVVDGGGSLR	78.9	537.0695	1072.1244
						2	DVDEINGCDIGVR	66.5	731.6575	1461.3004
2101*	CM02d_C3	Eukaryotic translation initiation	gi|3024017	75	19	1	ELVF**K**EDGQEYAQVLR	45.4	642.4005	1924.1797
		factor 1A				2	DYQDD**K**ADVILK	52.4	712.098	1422.1814
3101	CM02d_E5	heat shock protein	ROA_067935_0424_3607	120	22	2	ADLPGLK	39.2	357.4087	712.8028
		[homologous match]				1	**K**EEV**K**VEVEEGR	32.3	477.7563	1430.2471
						2	EEV**K**VEVEEGR	53.2	652.0981	1302.1816
4103	1504_F5	17.7 kDa heat shock protein	gi|1235898	238	26	2	ADLPGLK	39.7	357.3622	712.7098
						7	**K**EEV**K**VEVEEGR	79.9	715.8541	1429.6936
						5	EEV**K**VEVEEGR	58.7	652.0201	1302.0256
						5	VLQISGER	30.3	451.5257	901.0368
						5	AAMENGVLTVTVPK	32.4	723.6349	1445.2552
5001	CM02d_F5	stress and pathogenesis-related	gi|3901018	858	65	3	GVFTYESENTSVIPPAR	84.8	934.2761	1866.5376
		protein				13	AFVLDADNLIPK	30.5	659.2544	1316.4942
						2	AFVLDADNLIP**K**VAPQSIK	42.0	680.6581	2038.9525
						7	STETLEGDGGPGTIK	44.6	731.3245	1460.6344
						5	STETLEGDGGPGTI**K**K	66.3	795.6741	1589.3336
						3	**K**ITFGEGSQFK	60.5	414.6845	1241.0317
						4	ITFGEGSQFK	49.7	1113.4546	1112.4473
						1	ISYEI**K**IVASPDGGSLLK	25.6	630.7996	1889.377
						6	IVASPDGGSLLK	63.3	578.6285	1155.2424
						2	GNHEI**K**EEEVK	55.8	656.6091	1311.2036
						10	AVEAYLLAHPDAYN	47.7	774.1621	1546.3096
5002	1504_G5	superoxide dismutase	gi|38228697	263	40	1	HAGDLGNVNVGDDGTVSFTIIDK	72.8	782.4584	2344.3534
						4	QIPLCGPNSIIGR	53.1	713.6349	1425.2552
						4	AVVVHGDPDDLGK	55.5	661.611	1321.2074
						2	STGNAGGRIACGIIGLQG	45.4	851.7599	1701.5052
						4	IACGIIGLQG	45.6	1001.4746	1000.4673
5303*	1504_F7	triosephosphate isomerase	ABWP2_034459_1554_1107	250	52	3	VASPAQAQEVHFGLR	66.9	805.7179	1609.4212
						3	**K**WLQVNTSPEVAATTR	48.9	601.354	1801.0402
						1	IIYGGSVNGANCK	63.6	677.621	1353.2274
7107*	1504_B7	unknown, CBS domain protein	CCWP1_110480_2642_0865	274	53	2	VGDIMTEENK	49.3	576.5307	1151.0468
						2	VGDIMTEEN**K**LITVTLDTK	59.3	713.168	2136.4822
						1	LITVTLDTK	45.3	502.5542	1003.0938
						2	GMIGMVSIGDVVR	84.6	683.4301	1364.8456
						2	LNAFIQGGY	38.5	982.3546	981.3473
7110*	1504_C7	unknown, CBS domain protein	CCWP1_110480_2642_0865	230	53	2	VGDIMTEENK	56.2	576.5154	1151.0162
						2	VGDIMTEEN**K**LITVTLDTK	61.3	713.1682	2136.4828
						2	GMIGMVSIGDVVR	80.2	683.6461	1365.2776
						2	LNAFIQGGY	40.0	982.4321	981.4248
7203	CM02d_H3	none								
7408*	CM02d_A4	annexin	ACHS1n_159819_2640_1758	209	66	2	WTSSNQVLMEIACTR	84.0	906.6581	1811.3016
						2	SSHDLLLAR	37.8	506.6234	1011.2322
						1	SLEEDVAYHTTGDFR	28.8	580.6663	1738.9771
						2	YEGDEVNMTLAK	60.4	693.4765	1384.9384
7408*	CM02d_A4	isoflavone reductase like	ABWP1_026351_0296_1851	203	37	1	SKILIIGGTGYIGK	57.7	710.562	1419.1094
						2	ILIIGGTGYIGK	70.4	602.8907	1203.7668
						2	SGHPTFALVR	36.0	542.7687	1083.5228
						2	ESTVSDPVK	38.7	481.347	960.6794
7602*	CM02d_B4	dehydrin	ABWP1_045441_0451_3051	225	53	1	VFEKIPGAGNK	46.8	580.3038	1158.593
		[homologous match]				1	IPGAGNKDR	40.9	465.1174	928.2202
						2	D**R**VQGEQYR	41.0	576.0114	1150.0082
						1	GAMDKVFEK	30.4	521.1149	1040.2152
						2	IPGAGNKDK	43.2	450.2458	898.477
						2	DKVQGDQYR	34.7	555.0321	1108.0496
8002	CM02d_E4	none	none							
8104*	1504_C9	glycine-rich RNA binding protein	ABWP1_015278_0178_1196	100	24	2	AFSPYGEILESK	49.6	671.1248	1340.235
		[homologous match]				2	NITVNEAQSR	50.5	566.5034	1130.9922
8104*	CM02d_F4	glycine-rich RNA-binding protein	ABWP1_015278_0178_1196	97	25	2	AFSPYGEILESK	50.4	671.1497	1340.2848
		[homologous match]				2	GFGFVTFSNEK	46.6	617.2156	1232.4166
8202	1504_D9	none	none							
8303	1504_H8	none	none							
8309	1504_A9	none	none							
8309	CM02d_A2	none	none							
8506*	1504_F8	glyceraldehyde 3-phosphate	ROB_045015_1217_3381	176	57	2	AASFNIIPSSTGAAK	39.8	718.1123	1434.21
		dehydrogenase, cytosolic				2	VPTVDVSVVDLTVR	82.6	750.6729	1499.3312
						2	FGIVEGLMTTVHSITATQK	54.0	684.0022	2048.9848
8607	1504_E8	glyceraldehyde-3-phosphate	gi|120666	237	21	2	FGIVEGLMTTVHSITATQK	38.7	684.0699	2049.1879
		dehydrogenase, cytosolic				2	AASFNIIPSSTGAAK	47.5	718.1649	1434.3152
						2	VPTVDVSVVDLTVR	88.8	750.368	1498.7214
						4	AGIALNDNFVK	37.9	581.4758	1160.937
						2	LVSWYDNEWGYSTR	72.0	888.5875	1775.1604
8607	CM02d_C2	Ran binding protein	ABWP1_041027_0417_3079	146	33	4	LEEVAVTTGEEDETSILDLK	117.4	1096.213	2190.4114
						2	FDKDGNQWK	28.9	569.5167	1137.0188
9302*	1504_G8	germin-like/unknown/predicted	CCNHS_162322_3610_0519	282	48	3	IPGLNTLGVSLSR	85.5	663.9008	1325.787
						3	IDYAPGGLNPPHTHPR	77.0	872.1409	1742.2672
						2	SIKKGEIFVFPK	66.0	697.1452	1392.2758
						4	KGEIFVFPK	53.6	532.7322	1063.4498
9401	CM02d_B5	none								

^a^Identification of the NCBI protein match, or for EST peptide matches, the BLASTP identification of the EST. Mass spectrometry data and identifications have been submitted to the PRIDE database. See the field ‘gel spot identifier’ in the Identification Detail View to review individual spot spectra and identifications.

^b^NCBI number of the matching protein, or the Fagaceae genomics EST ID for peptided matching EST's.

^c^MASCOT score is a probability based score. Reported scores are sums of peptide identifications that are significant (except homologous where noted), and higher scores are equivalent to lower probability of an equal or better match occurring at random.

^d^Underline indicates an oxidized residue. Bold indicates a missed trypsin cleavage.

^e^Peptide mascot score for each peptide (or the highest match for multiple peptides).

^f^ * indicates spot with significant BBD effect and non-significant STAND and STANDxBBD interaction effect, and are the primary targets for biomarker development.

**Table 5:** Previously published stress response of proteins identified by MS/MS

**Category**	**Protein (or protein family)**	**Spot(s)**	**Quantification in healthy trees relative to diseased trees**	**Previously published stress response**	**References**
reactive oxygen species	polyphenol oxidase	109	up	wound, pathogen, insect	[17-19]
	glyceraldehyde-3-phosphate dehydrogenase	8506 8607, 1^st^ ID	up	hypersensitive response mimic, hydrogen peroxide, wounding	[20-23]
	annexin	7408, 1^st^ ID	up	oxidative stress	[24-26]
	thioredoxin	1004	down	oxidative stress	[27-30]
stress and pathogenesis related proteins	dehydrins	7602	up	drought, low temperature, freezing salinity, abscisic acid treatment	[31-33]
	isoflavone reductase-like protein	7408, 2^nd^ ID	up	lignification	[34]
	germin-like proteins	9302	down	pathogen, aluminum, wounding	[35-39]
transcription and translation	eukayrotic translation initiation factor (eif1)	2101	up	salt	[40,41]
	glycine-rich RNA binding protein	8104	up	hydrogen peroxide, flooding, cold, drought, salinity, abscisic acid treatment	[42,43]
	alpha nascent polypeptide associated complex 1 (αNAC1)	307	up	salt, systemic acquired resistance inducer	[44-46]
	Ran binding protein(RanBP)	8607, 2^nd^ ID		auxin treatment	[47]
unknown, or previously metabolic only	triose-phosphate isomerase (TPI)	5303	down	systemic acquired resistance inducer, abscisic acid treatment, pathogen	[46-49]
	CBS domain containing protein	7107, 7110	up, down	stress responsive	[50]

Many of the proteins with a BBD effect that were identified by MS/MS are previously published as stress responsive in other plant systems.

### Utility of the analysis to narrow the biomarker candidate pool

In order to illustrate the discriminatory power of our approach we have illustrated the spot set reduction strategy in Figure 3. Beginning with the 987 total protein spots identified, we show how at each step some spots are discarded from further consideration as a biomarker. The final set for continued biomarker considerations is eleven spots that have a BBD effect only (not confounded by stand effects or interactions between BBD and STAND) and are identified by their sequence homology.

## Discussion

### Challenges of proteomic investigation of forest trees

In general, protein extraction from plant tissue is technically challenging due to the high proportion of contaminants relative to the low concentration of protein. Proteomics in forest trees is further complicated by the complexity of working with trees as an experimental system due to factors such as their large size, long life cycle, and large genome [51]. In contrast to most proteomics studies conducted on model organisms, our subjects are wild, unrelated, mature trees selected from multiple stands. Like many forest trees, American beech is wind-pollinated and has a low self-pollination rate [52], resulting in high heterozygosity among trees within stands [53]. We selected trees from eight non-contiguous stands, further decreasing any chance of relatedness between trees across the study and likely increasing the number of alleles per locus sampled [54]. These factors lead to our study having a much higher degree of genetic complexity in the sampling units than is generally encountered in proteomics work where the use of inbred lines, clones, or pooling across genotypes is common. In addition, the multi-component nature of beech bark disease also adds to the complexity of protein patterns. Due to BBD having both an insect and a fungal component, both wound/insect and pathogen responsive genes are likely to be detected in diseased trees. In addition, BBD develops over a time scale of months or years, rather than the time course of days often studied in wound (insect), gene-for-gene, or viral pathosystems. BBD develops as a chronic disease, with significant associated bark damage including cracking, callous formation [1], and likely secondary local stress effects such as dehydration or nutrient and photosynthate transport disruptions. These bark stress factors may induce other, poorly understood sets of stress responses. The signalling pathways triggered by these multiple sources of stress are also likely interconnected through their regulatory pathways, which in other plant systems have been shown to be antagonistic, further adding to the complexity and variation of the response of the tree to the causal components of beech bark disease [55].

A high level of genetic diversity across geographical regions was previously reported in maritime pine [54]. In our study, genetic diversity across geographical regions is combined with the complexity of host responses to a dual pathogen disease system making the high number of spots identified in samples (up to 522 per tree, total of 987) not unexpected. Resolution of similar ranges of spot numbers (from 451 up to 753) have been previously reported in other plant species using similar supplies, equipment, and technical approaches [56-60]. Technical effects were significant for only six spots indicating that the observed differences between gels and samples were accounted for by the experimental design and analysis. Bark samples were anecdotally different for some visible features (such as chlorophyll content) that may vary by season, micro-site, or sample. Several spots that were not identified by tandem mass spectrometry had some (non-significant) homology to Fagaceae EST's, suggesting matching of unidentified peptides may be possible as additional woody plant, bark, and particularly American beech sequences become available. The low number of identified proteins in forest tree proteomic studies in general is a direct reflection of the lack of genomic DNA, EST and protein sequence data entries in public databases [51] for forest trees and for woody tissues.

The genetic complexity of the sample unit, the sampling across a wide geographic area, and the complexity of the BBD phenotype all contribute to possible protein differences between trees in the study. This complexity, especially combined with typical technical sources of variation, required careful study design and more elaborate statistical considerations than many proteomics studies. Identification of up to 101 protein spots unique to an individual tree emphasizes the genetic diversity captured in our study. One-hundred-twenty protein spots (22.5% of the matched spots) were identified as BBD significant despite the experimental complexity, so the experiment was effective at finding proteins of interest. We controlled the false discovery rate to 5% (using the q-value), so we would expect only six of the 120 proteins identified as differentially expressed to be erroneous.

### Sequenced spots have homology to known stress, insect, and pathogen related proteins in other plants

Most of the proteins identified by MS/MS in this study have homology to proteins known to be involved in stress responses in other plants (Table 5). The variety of biological responses to which these proteins are linked are consistent with the complexity of BBD. Since BBD has both an insect and a fungal component, it is not unexpected that both insect and pathogen related proteins would be found. BBD is also a long-term disease with both bark damage and significant healing as part of the physiology of the disease. Wounding, reactive oxygen species and drought responsive proteins are also expected. The variety of stress responses apparently influenced by BBD along with the identification of proteins involved both in transcription and translation control and basic metabolic responses, supports a model that beech trees have an active physiological response to BBD. These proteins are good targets for further research to understand the pathways involved in response to BBD.

The proteins identified in this study also expand on recent studies of the proteome of European Beech (*Fagus sylvatica* L.) [61-63]. While our study is the first publication of the proteome of bark tissue or any tissue from mature trees from *Fagus,* a few proteins identified in our study were also identified in other studies in *Fagus*. Of particular interest, Valcu et al. [63] identified triose-phosphate isomerase (TPI) as having a lower protein level in wounded leaves, while we found the protein to have higher expression in the bark of BBD diseased trees. Beech trees afflicted with beech bark disease would be responding to both scale feeding and pathogen infection (*Neonectria* spp.). The higher expression of TPI in diseased trees is consistent with the reports of higher expression of TPI in response to a fungal pathogen in *Brassica carinata*[48]. Further study of TPI to understand its role in defense in different tissues and different stages of beech bark disease infestation is certainly warranted.

### Biomarker candidates for further analysis

The long term goal of our research program is to identify broadly useful markers for BBD resistance that forest managers can use to plan for and mitigate BBD damage as it spreads to new regions and stands. A resistance biomarker could also be used to expedite the selection and breeding of scale-resistant trees in on-going tree improvement programs [10].

Twelve of the sequence identified spots (marked with an asterisk in both Table 3 and Table 4) showed a significant BBD effect and no effect of stand or BBDxStand interaction effect. Proteins different in healthy versus diseased trees regardless of stand (i.e. across stands) are the best biomarker candidates. These proteins are the most likely to be linked to resistance or susceptibility across broad geographic and genetic ranges, and so are the highest priority for follow-up study and biomarker development. Quantitation of these proteins in additional trees, especially in trees from other stands and regions, will be important in determining if these proteins can be used as biomarkers. Additional 2-DE gel studies, or development of antibody based (western blot, or ELISA) methods will facilitate this. RNA expression studies may also be helpful in understanding which proteins (genes) are the best markers for BBD resistant trees. An additional five spots that weren't identified by sequence homology fit into the same class and may be identified in the future as additional sequence becomes available. Often spots unidentified by MS/MS are dropped from further study. But given both the low amount of *Fagus* sequence available for comparison and the low amount of sequences in the database from bark tissues and forest trees in general, these spots are still good candidates for further study for biomarker development.

Proteins for which BBD effect is significant along with stand, interaction, or both require more careful interpretation, but may still become useful biomarkers. In combination with significant stand and BBD effects, a significant interaction effect means that the direction of association of a BBD effect (e.g. increased protein in healthy trees) is not consistent in the different stands. When STAND and BBD effects are both significant (but with a non-significant interaction effect), the protein may not be consistently distributed in American beech and may be present in only some of the stands managers wish to screen. Both of these groups of proteins are less attractive as biomarker candidates, but interpretation of them along with other higher priority candidate proteins may be insightful.

Most of the proteins identified by sequence have been found to be responsive to stress, insect, or pathogens in other plant systems, and are differentially expressed between the healthy and diseased trees. A small number of these proteins will be selected for further study, with priority given to those that are higher in the healthy trees and are predicted to be involved in insect resistance because of the known scale-insect resistance of the healthy trees and the requirement of scale feeding induced bark wounding to provide subsequent entryway for *Neonectria* infection [1,2,7]. Polyphenol oxidase and glyceraldehyde dehydrogenase both fall into this class and are considered high priority for further analysis. It will be important to continue validation of expression results using additional trees from the same stands, and additional stands from new geographic regions. In addition, the best biomarker protein may not be the one identified in this study, but rather a protein acting upstream in a response pathway, or regulating a response pathway(s). Further characterization of the biochemical pathways, and their induction through time, season, and spatially through the tree will be important. It is possible the proteins identified in this study will coincide with quantitative trait loci for scale resistance.

## Conclusions

American beech is an ecologically important species in many North American forests, only a portion of which are currently impacted by BBD [64]. Development of management options to reduce the economic losses and ecological costs of BBD are critically needed. This study has identified protein spots differentially expressed in the bark of healthy, scale-resistant trees and BBD-susceptible trees. This identification suggests that American beech has an active physiological response to BBD. Confirming this response is an important first step in understanding how BBD may progress physiologically and mechanistically in BBD susceptible trees, and how BBD resistance may be manifested. Additionally the results of this study should support and complement on-going strategies to find biomarkers for BBD resistance.

## Methods

### Selection of beech trees and collection of bark samples

Ten healthy trees were identified in seven stands in Fredericton, New Brunswick, Canada (Table 1). Healthy trees comprised only 5% of the beech trees in this area and were included in this study only if they were greater than 15 cm DBH [16]. All of these stands have been under attack by both *Cryptotoccus fagisuga* and *Neonectria spp.* since the early 1930’s [1] and would be considered an aftermath forest. After initial mortality waves, the remaining trees in an aftermath forest are primarily heavily cankered and a lower density persistent scale infestation is present in the stand. Diseased trees were selected along with healthy trees in five of the stands. A healthy tree (greater than 10 cm DBH) and a susceptible tree from Ludington, MI., USA were also included in this study. Beech scale is estimated to have been established in Ludington as early as 1990 and the presence of *Neonectria* was confirmed in 2001 [11]. At the time of tissue collection, 2004, this was considered a killing front. All diseased trees selected for this study showed visible signs of *Neonectria* infection such as cankers or the presence of perithecia and scale infestation.

The experimental sampling is unbalanced with respect to disease resistance because the primary interest is in resistant genotypes for breeding (more resistant trees are selected for grafting, testing, and planting). Modern statistical algorithms and computer power are sufficient to allow significant imbalance in experiments to be modelled, and we take advantage of this in our experimental design. All trees selected were tested for resistance to the beech scale insect in studies reported previously [16,65] and summarized in Table 1. These tests demonstrated that all the healthy trees were resistant to the beech scale insect and all of the diseased trees were susceptible to the beech scale insect.

Branches, 1–2 cm in diameter, were removed from the crown of each of the selected trees in October, 2006 in the New Brunswick stands and in September 2004 in Ludington, MI. Bark was peeled from the branches with a potato peeler and bark strips were placed in labelled 50mL falcon tubes, flash frozen and stored in liquid Nitrogen or a dry ice ethanol bath on site. Peeled bark collected from each tree was divided among three tubes and transferred to a −80°C freezer for storage either at the Natural Resources Canada Lab in Fredericton, New Brunswick, Canada or the US Forest Service (USFS) Lab at Delaware, Ohio, USA. In February of 2007, samples from New Brunswick, Canada were shipped overnight on dry ice to Delaware, OH, USA.

### Protein extraction

Protein was extracted according to Bona et al. [66] with minor modifications to account for the high soluble phenolic content of tree bark and phloem tissues. Bark tissue from each tree was combined with dry ice and ground to a course powder in a standard household coffee grinder and then transferred to a −80°C freezer. Three technical replicates were produced from the tissue from each tree. For each replicate, 2g of powdered tissue (after dry ice sublimated off) were combined with 2g of frozen polyvinyl-polypyrrolidone and 20mL of lysis buffer (as per Bona et al. [66] except that 1% Sigma Plant Proteinase inhibitor cocktail, P-9599, was used in place of 2% phenylmethylsulfonyl floride/dimethyl sulphoxide) and homogenized using a tissue homogenizer (Janke & Kunkel, IKA Labortecknik, Ultra-Turrax T25 with 18N tip). The resulting homogenate was centrifuged at 26,000g_n_ for 10 minutes at 4°C to pellet solids. The supernatant (generally 10 mL) was combined with 10 mL of tris(hydroxymethyl)aminomethane (Tris–HCl, pH 8.8) saturated phenol and mixed for one hour at room temperature. The phenolic phase was separated by centrifugation and rinsed with another 10 mL of lysis buffer, followed by further centrifugation to separate the phenolic phase. The final phenolic phase was recovered and proteins were precipitated by adding five volumes of methanol/0.1M ammonium acetate and incubating overnight at −20°C. Proteins were pelleted by centrifuging at 26,000g_n_ for 20 minutes and the resulting pellet rinsed three times with cold methanol, once with cold acetone, and dried under vacuum. The pellet was resolubilized in 450uL of resolubilization buffer (Biorad ReadyPrep sequential extraction reagent II (8M Urea, 4% 3-[(3-cholamidopropyl)dimethylamonio]-1-propanesulphonate (CHAPS), 40mM Tris, 0.2% Bio-Lyte 3/10 ampholytes) plus 1% tris-butyl phosphate (TBP, Sigma T-7567) and 1% plant proteinase inhibitor cocktail). Proteins were quantified (in triplicate) using the Biorad RC DC protein assay kit (Biorad 500–0118) microfuge tube assay protocol with the optional second wash. Protein quality was checked by running 40μg of protein on a denaturing polyacrylamide gel and staining with coomassie stain as per standard protocols (Biorad mini-protean-3 cell Instruction Manual).

### Two-dimensional electrophoresis (2-DE)

2-DE was conducted at the Plant Microbe Genomics Facility at The Ohio State University (OSU). Isoelectric focusing (IEF) was performed using 11cm pH 3–10 immobilized pH gradient strips (Biorad, 163–2014) in the Protean IEF Cell (Biorad). For quantitative gels, 100 μg of protein was mixed with rehydration buffer (7.0M Urea, 2.0M Thiourea, 2.0% w/v CHAPS, 2.0% w/v sulfo-betain 10 and focused for 55 kVH at 25°C. After IEF, the strips were treated according to the ReadyPrep Reduction-Alkylation kit (Biorad, 163–2090) which uses TBP for reduction, and iodoacetamide for alkylation. Second dimension separation was carried out on Criterion 8-16% Tris–HCl gels (Biorad 161–1394) using a Criterion Dodeca cell so that all eight gels in the replicate could be run in parallel. Gels were run at 200V for 60 minutes and then fixed for 30 minutes in a solution of 10% methanol and 6% acetic acid. Gels were then stained with 1x SYPRO-Ruby (Biorad, 170–3138) following manufacturer's instructions. Post staining, gels were de-stained for 1 hour in identical solution as that used for fixation. Preparative gels for spot cutting to recover proteins were prepared in the same way, except that 450 μg of protein was used per sample and gels were stained with Coomassie stain (Biorad, 161–0787) following manufacturer's instructions.

### Image analysis and quantification

Gels were imaged on a VersaDoc imager (Biorad), and the software program PDQuest (version 8.0, Biorad) was used to conduct the image analysis, spot identification and quantification. Gel images were digitally cropped along the outer edge to remove the molecular size marker and gel edges, and to standardize image size, but both pI fronts and the full size resolving area were retained.

The spot selection and gel matching were conducted in two stages, first a separate master gel was created for each tree by auto-matching the three replicate gels using the 'create experiment' dialog boxes of PDQuest. For these tree master gels, the spot detection and automated spot matching are conducted as part of the same procedure. For spot detection we used the spot detection wizard with vertical streak reduction on, and selecting the user chosen reference spot for small spot, faint spot, and large spot cluster from the same region of the gel for all gels. In addition we selected the local area regression method of normalization, which is proprietary but appears to be based on similar microarray normalization methods (see Quackenbush [67]). For spot matching, we defined no groups and spots were added to the master image only if present in two of three gels. Auto-matched spots were manually checked and corrected by dividing the gel area into 81 quadrants and hand marking landmark spots in each quadrant present in all three gels. All of the matches were hand checked based upon these landmark spots, and manual corrections to the spot detection and auto-matching were made, including removal of spots detected on the unresolved pI fronts and gel edges.

The second phase of image analysis was to create a 'compare experiments' analysis including all sixteen individual tree 'master gels' (CM02d was software selected as the base master gel). Automated matching was used to create the initial master file, then all matches were manually checked. Additional spots were added to the master manually if they were present in two or more tree masters. We applied the same hand check quality control as for individual tree masters (manually checking and correcting each of 81 quadrants using landmark spots) and applied the same normalization method (local area regression model option). Of note, we did not incorporate an additional scaling factor and the normalization method doesn’t scale the data, so the final spot quantities still have the original unit of counts.

Once the compare experiment master gel was fully checked, a quantitative dataset was created. The quantitative dataset was output from PDQuest using the function Report| Quantity Table Report, with the settings: all matched spots checked, configuration set to 'individual gels', missing spots set to 'estimate', and saturated spots set to 'estimate'. Spot quantities were estimated so that analysis options that require balanced and nonzero datasets could be used. PDQuest estimates saturated spots by fitting a Gaussian spot to the edges only and extrapolating the peak, then calculating the estimated volume from the extrapolated value. PDQuest estimates missing spots as the value of a minimum detectable spot. The resulting report contained spot quantities for all spots in the master gel across all 48 experimental gels. Graphical analysis of the spot quantities by spot were deemed sufficiently normally distributed to proceed with modelling.

To be sure the unmatched spots that are unique to one tree were not artifacts related to low spot intensity or variance in protein quantification making it difficult to match them, a random check of the intensity distribution of unmatched spots was conducted. Four trees were selected at random, then the spot intensity output for all spots on the tree master for these four trees was examined both visually by inspecting spot intensities in the gels and by comparing the distribution of intensities of the unique spots to the matched spots. Comparisons were made both graphically and by using summary statistics (mean, minimum, Q1, median, Q3, and maximum values).

### Experimental design and statistical analysis

The experiment included 16 trees, each of which had a location (stand) code and a disease condition code. Three replicate extractions (for three separate gels) were run per tree. For each replicate there were a total of 16 extractions, one per tree. Each protein extraction was assigned first to one of four extraction sets (three healthy and one diseased tree per set), then extraction sets paired to form gel sets (two gel sets per replicate). Samples in an extraction set were extracted in parallel, and gel sets were run in parallel for both IEF and polyacrylamide gel separation. Hence, each extraction had a full list of factors assigned: tree, stand, disease state, replicate, extraction set, gel set. The full dataset included these factors and spot quantities for each spot on the master gel for each of the 48 gels (see additional files: Additional file 2, full study design; Additional file 3, full set of spot quantities). Calculated spot quantities were from the normalized gel images, and were evaluated and determined to need no additional transformation. An ANOVA approach to statistical analysis was used to so that multiple effects and interactions could be included in the same model to better control error variance, and because the other biological effects (i.e. stand and its interaction with BBD) will be informative in selecting proteins for future study.

Statistical analysis was generated using SAS® software version 9.2 of the SAS system for Windows, copyright 2002–2008 SAS Institute Inc. SAS and all other SAS Institute Inc. products or service names are registered trademarks or trademarks of SAS Institute, Cary, NC, USA.

A series of tests were used to categorize each protein spot, to arrive at a list of candidate spots for further analysis (summarized in Figure 3). The first phase of the analysis sought to exclude constitutive proteins that did not differ between any trees, and assess the significance of the technical factors. The following model was fit using the General Linear Model procedure of SAS:

$$\text{intensity}=\mu +t_{j}+g_{k\left(1\right)}+\varepsilon_{\text{ijk}\left(1\right)}$$

where μ is an overall average, t_j_ is the effect of the j^th^ tree (tree effect), g_k(l)_ is the effect of the l^th^ extraction set nested within the k^th^ gel set (technical effect), and ε_ijk(l)_ is a random error term. The model was fit for each spot, and the test of significant effects computed using the type III sums of squares. Model fit was evaluated by verifying that the overall model fit had a significant F value (p-value < 0.05) and by examination of standardized residuals (especially by plotting against the levels of effects). For each model, careful assessment of residual plots confirmed model assumptions about error distribution and equal variances were sufficiently met (i.e. the spot quantities did not deviate from the assumed normal distribution enough to warrant additional transformation). Degrees of freedom (df) were the same for each spot model: tree effect has 15 df, technical effect had 11 df, and error df = 21 (total df=47). For the technical effect, a Bonferroni adjustment was used to determine significance level, but for the tree effect a p<= 0.05 was considered significant. This permissive cut off is appropriate since the goal of the test was to eliminate constitutive proteins (and reduce the physical size of the dataset to ease computations) and because a false acceptance of the null is more problematic than a false rejection at this point in the analysis. Any spots that are not significantly different in at least one tree (tree effect p<0.05) were dropped from the dataset. Technical effects were found to be not significant and were dropped from further analysis for all but six spots that were dropped from the dataset.

The second phase of the analysis was designed to determine how spots differed between trees. Technical factors were dropped and stand and disease state factors were added (there were insufficient degrees of freedom to analyse technical and biological factors in the same model, partly due to imbalance in the number of trees selected per stand). The following model was fit using the General Linear Model procedure of SAS:

$$\text{intensity}=\mu +s_{j}+d_{k}+{\text{sd}}_{\text{jk}}+\varepsilon_{\text{ijk}}$$

where μ is an overall average, s_j_ is the effect of the j^th^ stand (stand effect), d_k_ is the effect of the k^th^ disease state (BBD effect), sd_jk_ is the interaction effect of stand and disease state, and ε_ijk_ is a random error term. Model fit was evaluated by verifying that the overall model fit had a significant F value (p-value < 0.05) and by examination of standardized residuals (especially by plotting against the levels of effects). For each model, careful assessment of residual plots confirmed model assumptions about error distribution and equal variances were sufficiently met (i.e. the spot quantities did not deviate from the assumed normal distribution enough to warrant additional transformation). Degrees of freedom were the same for each spot model: BBD effect has 1df, STAND effect has 7 df, BBDxSTAND interaction effect has 5 df, and error has 34 df (total df=47). The model was fit for each spot, and the test of significant effects computed using the type III sums of squares. Interaction effects and stand effect were tested using the conservative Bonferroni correction. For the BBD effect, p-values from the tests were output to a new dataset and the package 'qvalue' (q-value 1.1) for the statistical program R (R-2.4.0-win32, [68]) was used to compute the associated q-value for each test. Significance was determined using q-values while controlling the false discovery rate at 5% [69]. False discovery rate controls the percentage of null hypothesis rejected in error (false positives) rather than the overall error rate, and is an accepted and typical statistical analysis for large genomic and proteomic datasets [70].

### Spot selection and cutting

All spots with a significant effect for the disease state factor were considered for spot cutting and sequencing. Spot quantities were evaluated in all of the trees and trees ranked as the best trees were those having the most BBD significant spots at the highest spot densities. The two top trees were used for preparative gels and spot cutting. All BBD significant spots in the two selected trees were evaluated on the gel images to determine if the spot could be excised cleanly and was sufficiently intense to support sequencing. Spots were excised from the preparative gels at the PMGF using the Protean 2-D spot cutter (Biorad Laboratories). Several constitutive spots were also selected as sequencing reference spots. High resolution pre-cut and post-cut images of preparative gels were captured on the VersaDoc imager and evaluated for quality control. Only protein spots that were cleanly excised and had no evidence of contamination from adjacent spots were sent for MS/MS analysis.

### Mass spectrometry

Mass spectrometry was performed at the OSU Campus Chemical Instrumentation Center. Gel pieces were washed twice in 50% methanol/5% acetic acid for one hour each, followed by dehydration in acetonitrile. Cysteines were reduced by rehydrating and incubating in dithiothreitol (DTT) solution (5mg/mL in 100 mM ammonium bicarbonate) for 30 minutes. Cysteins were alkylated by the addition of 15mg/mL iodoacetamide in 100 mM ammonium bicarbonate solution, and incubation in the dark for 30 min. The gel cores were washed again with cycles of acetonitrile and ammonium bicarbonate (100mM) in 5 min increments, then dried under vacuum. Protein was digested in Multiscreen Solvinert Filter Plates from Millipore (Bedford, MA) with sequencing grade modified trypsin (Promega, Madison WI) overnight. The peptides were extracted from the polyacrylamide by washing several times with 50% acetonitrile and 5% formic acid, pooled, and concentrated under vacuum to ~30 uL.

Capillary-liquid chromatography-nanospray tandem mass spectrometry (Nano-LC/MS/MS) was performed on a Thermo Finnigan LTQ mass spectrometer equipped with a nanospray source operated in positive ion mode. The LC system was an UltiMate™ 3000 system from Dionex (Sunnyvale, CA). Five microliters of each sample were first injected on to the micro-Precolumn Cartridge (Dionex, Sunnyvale, CA), and washed with 50 mM acetic acid. The injector port was switched to inject and the peptides were eluted off of the trap onto the column. A 5 cm 75 μm ID ProteoPep II C18 column (New Objective, Inc. Woburn, MA) packed directly in the nanospray tip was used for chromatographic separations. Peptides were eluted directly off the column into the LTQ system using a gradient of 2-80% acetonitrile over 45 minutes, with a flow rate of 300 nl/min and total run time was 65 minutes. The MS/MS was acquired using a nanospray source operated with a spray voltage of 3 KV and a capillary temperature of 200°C. The analysis was programmed for a full scan recorded between 350 – 2000 Da, and a MS/MS scan to generate product ion spectra to determine amino acid sequence in consecutive instrument scans of the ten most abundant peaks in the spectrum. The CID fragmentation energy was set to 35%. Dynamic exclusion is enabled with a repeat count of 30s, exclusion duration of 350s and a low mass width of 0.5 Da and high mass width of 1.50 Da.

### Sequence data processing and matching

Sequence information from the MS/MS data were searched using Mascot Daemon (version 2.2.1 Matrix Scientific, Boston, MA) [71] against several databases (detailed below). The search parameters were: mass accuracy of the precursor ions = 2.0, fragment mass accuracy = 0.5 Da, considered (variable) modifications = methionine oxidation and carbamidomethyl cysteine, missed cleavages = 2–4. Due to the low representation of woody plant and bark tissue sequences in the databases, the search was conducted against several databases. Searching against the full SwissProt database version 54.1 (283454 sequences; 104030551 residues) was unproductive (only procedural peptides identified, data not shown). A second search was conducted restricting the search set to taxon Viridiplantae (version, sequences, residues). The Fagaceae genomics project [72] has constructed EST libraries from American Beech, Red Oak (*Quercus rubra* L*.*), White Oak (*Quercus alba* L.), American chestnut (*Castanea dentata* (Marsh.) Borkh) and Chinese chestnut (*Castanea mollissima* Blume) including libraries constructed from both healthy and diseased stem tissues. Twenty-four individual EST libraries (#13696, 8-21-2009, 10691208 sequences; 751178460 residues) were compiled into a custom database and searched. Peptide matches were checked manually and only those identifications with a Mascot score of 50 or higher and having two or more unique peptides of five or more residues were accepted. For EST matches, peptides were matched to EST's (by the same criteria), then EST's searched against GenBank (BLASTP, default settings [73] to obtain protein identifications. Analysis data is available in the PRIDE database [74,75] under the accession numbers 17706. The data was converted using the PRIDE Converter [76,77].

## Abbreviations

2-DE: 2 Dimensional Electrophoresis; ANOVA: Analysis Of Variance; BBD: Beech Bark Disease, or its effect in a statistical model; CHAPS: 3-[(3-Cholamidopropyl)Dimethylamonio]-1-Propanesulphonate; DF: Degrees of Freedom in a statistical model or test; IEF: Iso-Electric Focusing; INT: The Interaction Effect (Bbdxstand) In Statistical Models; OSU: The Ohio State University; STAND: The Effect Of Stand In The Statistical Models; TBP: Tris-Butyl Phosphate; Tris–HCl: Tris(Hydroxymethyl)Aminomethane.

## Competing interests

We would like to acknowledge and declare several funding sources: Parks Canada Ecological Integrity Innovation and Leadership Fund, Fundy Model Forest, Canadian Forest Service, Northern Research Station of the US Forest Service, and the Special Technology Development Program of the Northeast Area State and Private Forestry, US Forest Service.

## Authors' contributions

MEM carried out molecular experiments, statistical analysis, and drafted the manuscript under the supervision of JLK who helped design the study and draft the manuscript. JLK, JL and MK developed the study concept and carried out field and artificial infestation experiments. All authors read and approved the final manuscript.

## Supplementary Material

Additional file 1 **p-values from ANOVA models.** Complete set of p-values under-laying Table 3 including the initial p-value and the computed q-values and determination of significance based on q-value or Bonferroni correction. Click here for file

Additional file 2 **Full experimental design.** The full design of the experiment by gel, including randomization to extraction batch and gel batch. Click here for file

Additional file 3 **Full set of spot quantities.** Spread sheet of the full spot quantifications for 48 gels showing the value of counts for each spot on each gel. Click here for file
